# Correlation between Students’ Self-Efficacy and Teachers’ Educational Leadership Style in Iranian Midwifery Students

**DOI:** 10.5539/gjhs.v8n7p260

**Published:** 2015-12-16

**Authors:** Zohreh Sohrabi, Masoomeh Kheirkhah, Elahe Sadegi Sahebzad, Seyedehsahel Rasoulighasemlouei, Siamak Khavandi

**Affiliations:** 1 School of Medicine, Iran University Medical Science, Tehran, Iran; 2 School of Medicine, Faculty Member Nursing & Midwifery School, Iran University Medical Science, Tehran, Iran; 3 School of Nursing and Midwifery, Iran University Medical Science, Mashhad, Iran; 4 School of Medicine, Tehran University Medical Science, Tehran, Iran

**Keywords:** self-efficacy, teacher, Leadership style, midwifery

## Abstract

**Background:**

Self-efficacy is believe in and feeling of ability to complete work. One of these factors is educational teachers’ role. This study aimed to determine relationship between teachers’ leadership style and students’ self-efficacy in midwifery students.

**Method:**

This Study is a cross sectional correlation study. Sampling was conducted in midwifery students in Bachelor Science degree in 2013. Data collection tools were multi leadership questionnaire and self-efficacy clinical performance. After explaining the goals of study, 97 students completed the questionnaire. Scoring the questionnaire was based on a Liker’s scale (0-5). Data were analyzed by SPSS 16. Correlation coefficient test was adopted to investigate the relationship and p value was considered 0.05.

**Result:**

Mean of self-efficacy scores were 116.12 (24.66.). In 53.3% of the cases, self-efficacy was good, in 42.2% moderate and in 4.3%, it was bad. The majority of the students (88.9%) reported that their teachers had an idealized style in leadership. About 94.6% of the students with good self- efficacy believed that their teachers’ leadership style was transformational style. There was a significant correlation between self- efficacy and leadership style (p<0.05).

**Conclusions:**

Results showed that transformational style is appropriate for midwifery teachers.

## 1. Introduction

Self-efficiency is defined as the individual’s belief and faith in their capabilities to do the responsibility and adapt with surrounding environment, as Bandura 1997 believes self-efficiency is evaluated with the individual’s judgment of their ability to adapt with the environmental demands. Such individuals perform more efficiently in stressful environments, apply a more effective strategy to deal with the challenges, and have more job satisfaction (Nielsen & Daniels, 2012). Self–efficacy is improved by an increase in knowledge and skills (Cohen & Cragin, 2010), and it gives individuals the ability to organize the activities and leads to motivation formation and more ability in dealing with the things (Lauder, Holland, & Roxburgh, 2008). Unsuccessful accomplishment of the jobs results from self-inefficacy in efficient other issues. Individuals’ judgments about their own function are originated from their self- efficacy (Modern Educational Psychology, 2013). The students with higher self- efficacy have more self- organization skills and use newer strategies of learning. In addition, they use their time more efficiently and do any necessary adapting activity to progress their learning (Laschinger & Tresolini, 1999). Self-efficacy helps students’ feel empowered in clinical psychology to accept challenges of their role in clinical practices. It is a good indicator for predicting students’ performance in clinical environments. Feeling that “I can” is associated with the feeling of mastery over the individual’s dealings with the environment (Masoudi Alavi, 2014).

Students’ verbal approval and providing a positive and helpful feedback is associated with an enhancement in their self-efficiency (Gibbon, 2010). There is a great emphasis on teaching – learning management, as the core of educational organizations activities regard to instructional leadership or learning based on it. Educational leadership is among the factors, effective on learners’ learning that can fulfill educational goals. Society needs trained workforce to give services and proceed the major goals of a country and expects the faculties to prepare the university students to do this important issue.

Academic members have a great duty to educational standards administration and training the students (Bush, 2010). Universities are the main background to provide the society with efficient human resources to fulfill its needs. In this direction, the universities of medical sciences have an important mission in professional role development (9). In order to progress this in students, it is recommended they perform clinical practices under professors’ supervision in order to gain required ability in such practices independently, and such a chance to practice and experience this should be given to them under their supervision. Moreover, professors should provide them with a chance to gain clinical experience and give them effective feedbacks constantly (Masoudi Alavi, 2014). Educational administration is the challenge that when the gap between the science and practice is noted, then, putting the education into the practice for enhancing the learners is vital and recommended in clinical practices (Melina, Burgess, & Falkman, 2013). The process of teaching and learning should be in the way to train responsible students with the ability of critical thinking, and such students should acquire the correct and disciplined feedback from their professors (Polard & Wild, 2014).

Among medical sciences graduates, despite cognitive goals, the final goal is on obtaining clinical skills, obtaining knowledge and appropriate attitude, and acquiring the needed experience to play an efficient and successful role in a profession and specific occupation (Nasiriani, Farnia, Salimi, Shahbazi, & Motavasselian, 2006). In health Department, clinical and educational administration is extremely crucial for the importance of care providing with good quality. These leaders should apply supportive leadership so that students and other staff members have sufficient motivation for care providing. The absence of communication skills in group work impairment and unsupportive leadership are the most important reasons of low quality care and staff’s request to move another workplace (Shirazi, Mirmoosavi, Alavinia, Zamanian, Fathollahbeigi, & Masiello, 2014). Leaders through evaluating their followers’ interests improve their-awareness and commitment, reaching to the preset goals and help the group to be improved. Such leaders promote the potential efficiency in the group through influencing the individuals’ knowledge and help to understand the mastery of the self-efficiency feeling (Nielsen & Daniels, 2012).

Midwives act as one of the members of health providing team in provision of quality care. Midwifery educational programs facilitate experience-based learning and train professional midwives with professional commitment who are aware of women’s and midwifery issues. The midwives should be capable of managing maternity health centers in proper and efficient manner and making a decision in pregnancy and delivery. Education and training of such students is a responsibility for the experienced academic members and educators of this course. Experienced, skillful and conducting teachers facilitate grammar of learning rather than just acting as an information resource (13). Overall, educational leaders play an essential role in defining goals, outlook, strategy, environment and work motivation, as national and international evidences in hygiene emphasize on their crucial role in improving care. However, one approach to leadership does not work properly in all situations, and leaders should adapt their approach with different situations, which is a response to frequent changes in educational and organizational situations. Transactional and transformational leadership are common approaches in leadership. Transactional leadership leaders for reaching their goals apply reward and punishment system, while transformational leaders emphasize on transforming the system shape by focusing on supportive behavior. There are many known benefits for this approach to leadership, such as job satisfaction, improving organizational efficiency and reducing the subordinates’ stress (Rosemary, 1979). While the educational leaders care about the professional concerns of students and can sharply influence on individual and professional? occupational promotion by providing specific educational materials and evaluating and constant supervision of students (Erchul & Raven, 2001). Regarding the importance of midwifery in mother’s and child’s health as a half of the society, regarding that there has not been any conducted study of midwifery professors’ leadership method and approach in theoretical and empirical education in midwifery and nursery universities and faculties and regarding that midwifery and nursery universities in Iran have a plethora of problems in theoretical and empirical education throughout the entire country that are even in big universities, improving the education quality and effective learning of students are the educational targets of those involved in educating this field.

## 2. Materials and Methods

This is a cross- sectional and correlational study, aimed to determine correlation between teachers’ leadership style and students’ self-efficacy in Nursing and Midwifery School in Tehran University in 2013.

Sampling was convenient and comprised of all term 6 and 8 midwifery students of Tehran University of Medical Sciences. With consideration of 10% subject drop, 97 students completed the questionnaires. Data collection tool was a 37- item questionnaire of self- efficacy in midwifery clinical function, designed by use of a self- efficacy tool made by Cheraghi (2009). Content and face validities were established by the academic members in Tehran nursing and midwifery school. Its reliability was determined by test retest (r=0.86). This scale was scored by a five- point Likert’s scale including absolutely disagree, disagree, no idea, agree and absolutely agree. Absolutely disagree was pointed zero and absolutely agree was pointed four. The students with scores 0-49 were categorized in low self- efficacy; 50-99, in moderate and 100-148 were categorized in high self- efficacy group. Multi Leadership Questionnaire including 36 items with a five- point Likert’s scale was adopted to investigate the Leadership style. This questionnaire was prepared by Bass and Avilio (1994) and has been frequently revised up to now. Its second version was adopted in the present study (Bass & Avilio, 2000). For its validity, indications and viewpoints of 10 experts of management sciences were considered in final draft of the questionnaire. For its reliability, the questionnaires were distributed among 30 subjects in the studied population and were collected after completion. After a 15- day interval, they were distributed among the same respondents again.

Correlation between questionnaire scores was calculated and test retest index of multi leadership questionnaire was obtained 0.721. Its internal consistency was also calculated by Cronbach alpha (α=0.90). Each item was scored based on a five – point Likert’s scale from never (score one) to always (score five). Items 2, 6,8, 9, 10, 13, 14, 15, 18, 19, 21, 23, 25, 26, 29, 30, 31, 32, 34 and 36 were associated to transformational leadership style and items 1, 4, 11, 16, 22, 24, 27 and 35 were associated to transactional and items 3, 5, 7, 12, 17, 20, 28 and 33 were for passive – avoidant leadership. The criterion to define educators’ leadership style is the total scores that students give to their educators based on aforementioned leadership styles. Any of the above- mentioned items collection that gets the highest score is considered as the dominant educators’ leadership style.

Transformational leadership style includes: Idealized Charisma (items 10, 18, 21, 25); Idealized behavior (items 6, 10, 23, 24); Inspirational motivation (items 9, 13, 26, 36); Stimulation of creative thinking (items 2, 8, 30, 32) and Client-centered attention (items 15, 19, 29, 31). Transactional leadership style includes: Appropriate reward (items 1, 11, 16, 35); Management based on active exception (items 4, 22, 24, 27). Passive - avoidant leadership style includes: Management based on passive exception (items 3, 12, 17, 20) and Non- intervention (items 5, 7, 28, 33). This questionnaire was given to the academic members of Nursing and Midwifery school in both Persian and English versions, and its validity was confirmed. Its reliability was determined by Bahadori et al. (2012) through test re- test method(r= 0.84). Descriptive, and inferential statistical tests such as Kolmogorov –Smirnov test (data normal distribution test) and Pearson correlation coefficient (to investigate variables associations) were adopted to analyze the data in SPSS16.

## 3. Results & Discussion

In the present study, there were 97 midwifery students with mean age of 20 years of whom 47 (49%) were students of term 6, and 50(51.4%) were students of term 8.

Mean (SD) score of students’ self- efficacy was 123 (24.66) (Min 67 VS Max 164). There was no association between educational semesters and self – efficacy scores (p=0.647). Self-efficacy score was evaluated as good in 52 (53.3%) students; moderate in 41 (42.2%) and poor in 4 (4.3%) of the students. Based on students’ viewpoints, 68 (69.6%) of the teachers had idealized behavior style, 2 (2.2%) had idealized Charisma, 3 (3.3%) had Inspirational motivation; 7 (6.7%) creative thinking transactional and 5(4.4%) had client – centered justifiable leadership style, 7 (6.7%) had transactional and 5 (4.4%) had passive – avoidant leadership style. In other worlds, 86 (88.9%) of the students believed that clinical midwifery teachers and educator had transformational leadership style and 7 (6.7%) and 4 (4.4%) had transactional and passive - avoidant leadership respectively. About 81 (84.21%) of the students believed that the teachers had idealized leadership style, and obtained a good self- efficacy score. Meanwhile, 66.6% of the students believed the teachers’ leadership style was passive and obtained a low self – efficacy score. About 42 (43.3%) of the students with moderate self- efficacy score believed their teachers’ leadership style was idealized. To investigate the correction and association between teachers’ leadership style and students’ self-efficacy, firstly Kolmogorov – Smirnov test was conducted and revealed that the data had a normal distribution. Then, Spearman and Pearson correlation co-efficient were adopted to investigate the association. There was a significant association between transformational leadership style and self-efficacy scores (p=0.001, r=0.55).

There was a significant association between transactional and self-efficacy (P=0.001, r=0.584), but there was no significant association between passive - avoidant leadership and self-efficacy (P=0.27). Man- Whitney test showed a significant association between leadership style and students’ self-efficacy (P=0.004, Z=3.086) (Table 1).

**Table 1:** Frequency distribution of the association between midwifery teachers’ leadership style based on midwifery students’ self-efficacy

self- efficacy leadership style			Good	Moderate	Poor	Total
Transformational	Idealized charisma	No	0	2	1	P=0.001
		%	0	2.06	1.03	R=0.561
	Idealized Behavior	No	55	13	0	P=0.001
		%	56.70	13.40	0	R=0.583
	Inspirational motivation	No	2	1	0	P=0.001
		%	2.06	1.03	0	R=0.421
	Creative thinking	No	2	5	0	P=0.001
		%	2.06	5.15	0	R=0.584
	Appropriate reward	NO	0	5	0	P=0.001
		%	0	5.15	0	R=0.601
Transactional Leadership	Active exception-based Leadership	No	2	3	2	P=0.001
		%	2.06	3.09	2.06	R=0.486
Passive-avoidant leadership	Passive-avoidant based leadership	No	0	5	0	P=0.27
		%	0	5.15	0	R=0.263

Self-efficiency is one of vital elements of Bandura’s social cognitive theory in learning, motivation and reaching academic goals (Anthony & Artino, 2012). Bandura considers self-efficiency as personal judgment about their ability to organize and perform for reaching the designed performance, which is not essentially the ability to perform a specific task in a certain field (19). In the recent study, the relationship between self-efficiency and leadership approaches, especially transformational leadership, is shown; however, this does not mean the ability to perform a specific task and there should be more research in terms of students’ ability in performing tasks after graduation. However, the recent studies in the past twenty years have shown that the self-efficiency is the most powerful factor for predicting students’ performance (Robbins et al., 2004) and spreading it is one of the university professors’ educational goals (Bandura, 1986). Also, studies show undergraduate students are under pressure due to learning and a lot of frequent tasks; thus, they need more facilitation in learning. If professors act to facilitate them and guide students, it increase their motivation, as a meaningful relationship between the interaction between teachers with their students and their role in their students’ self-efficiency is reported when facing problems in academic environment. Supportive behavior and proper communication with students increases sympathy and plays an essential role in the proper creation of their self-concept. Such students try to reach solutions according to plan (Charkhabi, Azizi Abarghuei, & Hayat, 2013). Leadership has been known as a vital factor in identifying goals, outlook and strategy in the workplace and increases motivation (Shirazi et al., 2014). The results of Rasooli’s study (2009) showed an effective and proper relationship between leader and their followers increases job efficiency and inner, outer and public satisfaction (Rasouli, 2009). Self-efficiency is a multi-dimensional understanding and several factors, like personal characteristics, individuals’ self-esteem and environmental factors, such as social support, are influential in self-efficiency. Encouragement is one of the social supports, which is essential in improving self-esteem, self-efficiency and self-management. Study results have shown interference with self-management is associated with reducing stress and emotional reactions, which increases self-efficiency and improves life quality (Hemmati Maslakpak & Raiesi, 2014).

Ghafoari et al in which an association was observed between teachers’ leadership style and students’ self-efficacy (Ghafoori, Ganjavi, & Dehghan, 2008). Borang (2008), in a study on students’ self-efficacy score in Azad University, Khorasgan branch, showed that most of the students evaluated their self-efficacy score as moderate (Borang & Amin Yazdi, 2008) On the other hand, a direct significant association was observed between passive leadership and students’ evaluation of poor self-efficacy. Meanwhile, there was no significant difference between students’ semester and their self-efficacy scores (p<0.647), which can show no effect of students’ semester on their self-efficacy, as the students measured their capability and self-efficacy based on the goals that had been already set, and their education process.

Leaders with transformational style can proceed the organizations toward future and facilitate appropriate changes (Borang & Amin Yazdi, 2008), Continuous education, providing the opportunity to improve in occupation and encouragement are the effective solutions. In case of educational leaders’ emphasis on occupational concerns, it will be possible to witness the training of students who can improve the healthiness of the community after graduation if the required educational materials are provided and if the supervision and evaluation are constant. Hence, applying new teaching approaches along with a proper leadership method can enhance students’ self-efficiency (Banihashemiyan, Golestan Jahromi, Gpk, & Sharafi, 2011). It should be noted that almost 10% of the undergraduate midwifery students in terms 6 and 8 did not attend the present study. In addition, the students’ mental and psychological status during completion of the questionnaires, despite confidentiality of the data, was out of researcher’s control. There is an association between teachers’ leadership style and students’ self-efficacy. This association is mostly observed in transformational leadership style, which leads to more students’ self-efficacy. Therefore, through bolding workshops, the teachers can be familiarized with this style and take steps toward improvement of students’ self-efficacy.
